# Supplementary material for: The Role of Pea (Pisum sativum) Seeds in Transmission of Entero-Aggregative Escherichia coli to Growing Plants
Source: Microorganisms. 2020 Aug 21;8(9):1271. doi: 10.3390/microorganisms8091271 (PMC7565074; doi:10.3390/microorganisms8091271)
Supplement: Supplementary file 1 [file microorganisms-08-01271-s001.pdf]

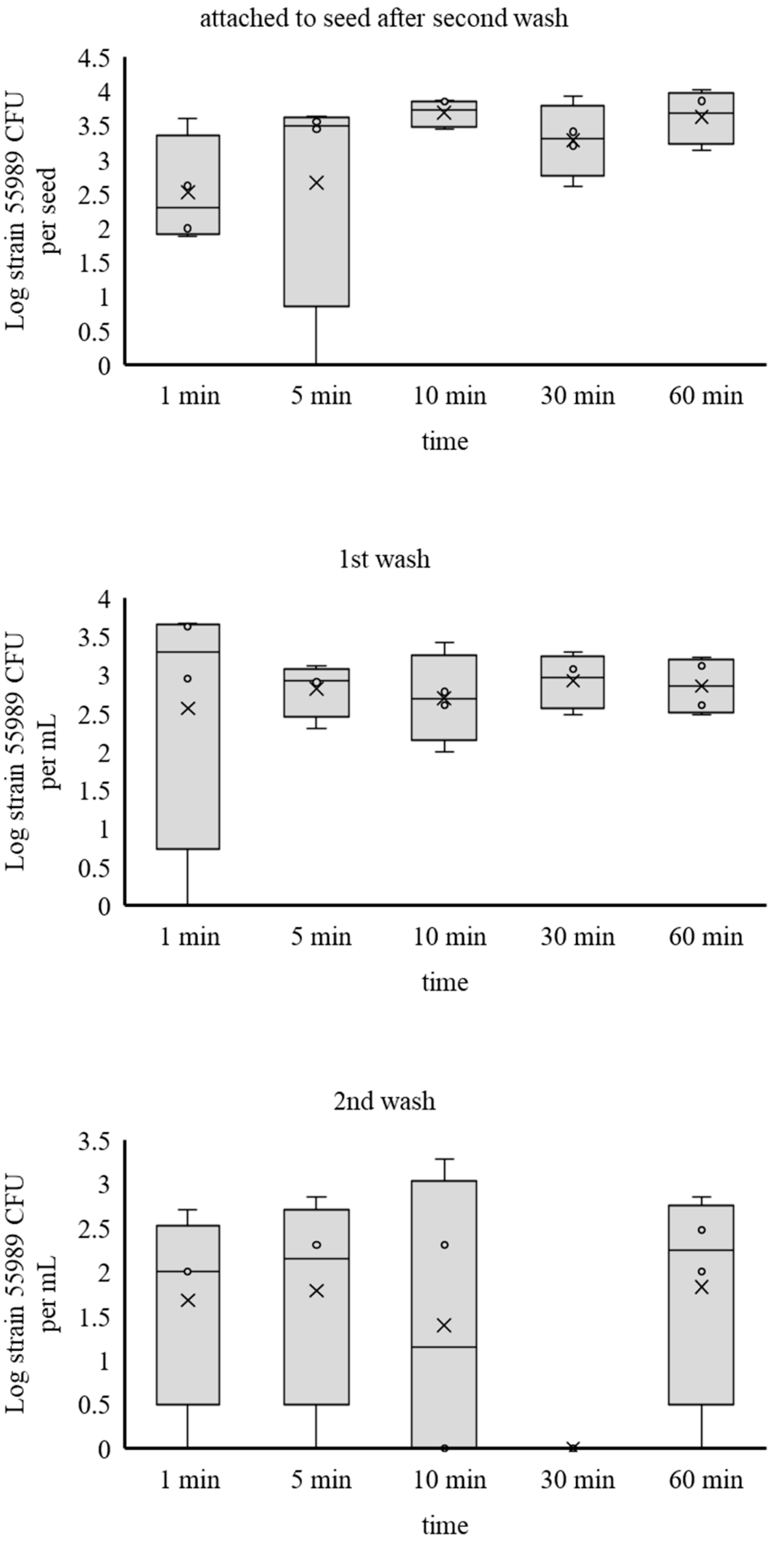

Figure S1. Recovery of strain 55989 CFUs from washed pea seeds (A) and from the first (B) and second (C) wash solutions Seeds were submerged for different time periods in a strain 55989 cell suspension.

.

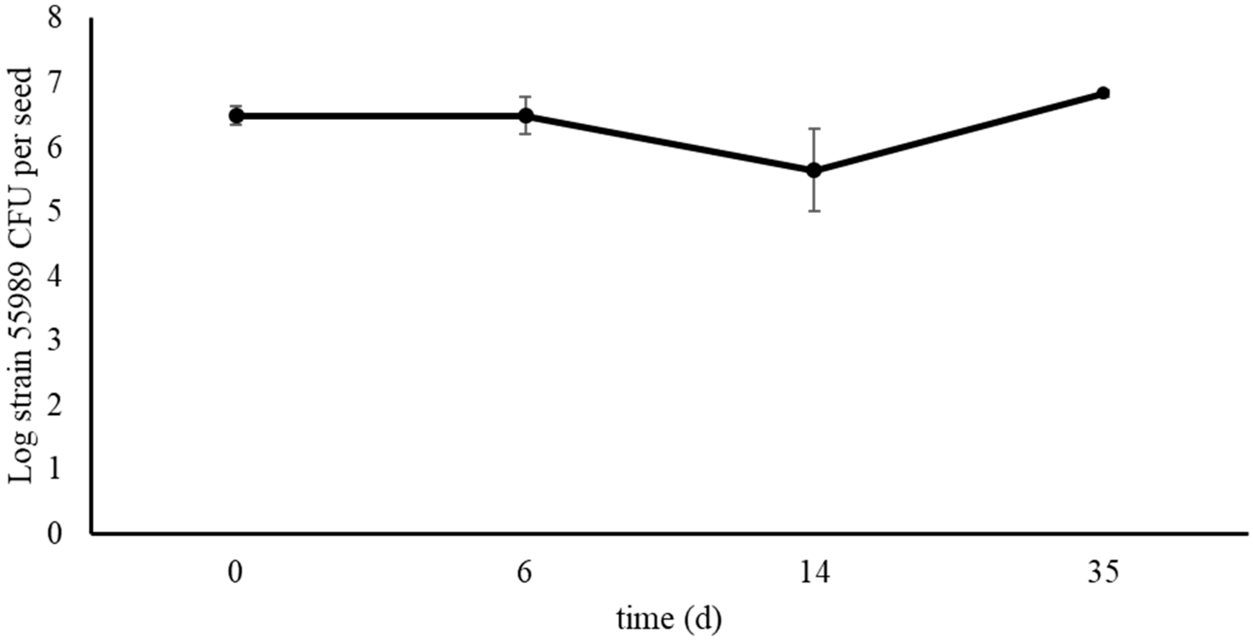

Figure S2. Persistence of strain 55989 cells on pea seeds. Seeds were submerged in a strain 55989 cell suspension, washed, dried and stored for 35 d at 4°C.
